# The Role of Connexin 43 and Pannexin 1 During Acute Inflammation

**DOI:** 10.3389/fphys.2020.594097

**Published:** 2020-10-29

**Authors:** Petra Kameritsch, Kristin Pogoda

**Affiliations:** ^1^Institute of Cardiovascular Physiology and Pathophysiology, Biomedical Center, Ludwig-Maximilians-University Munich, Munich, Germany; ^2^Walter Brendel Center of Experimental Medicine, University Hospital, Ludwig-Maximilians-University Munich, Munich, Germany; ^3^Medical Faculty, Department of Physiology, Augsburg University, Augsburg, Germany

**Keywords:** hemichannel, leukocyte, connexin, gap junction, acute inflammation, endothelial cells, ATP

## Abstract

During acute inflammation, the recruitment of leukocytes from the blood stream into the inflamed tissue is a well-described mechanism encompassing the interaction of endothelial cells with leukocytes allowing leukocytes to reach the site of tissue injury or infection where they can fulfill their function such as phagocytosis. This process requires a fine-tuned regulation of a plethora of signaling cascades, which are still incompletely understood. Here, connexin 43 (Cx43) and pannexin 1 (Panx1) are known to be pivotal for the correct communication of endothelial cells with leukocytes. Pharmacological as well as genetic approaches provide evidence that endothelial Cx43-hemichannels and Panx1-channels release signaling molecules including ATP and thereby regulate vessel function and permeability as well as the recruitment of leukocytes during acute inflammation. Furthermore, Cx43 hemichannels and Panx1-channels in leukocytes release signaling molecules and can mediate the activation and function of leukocytes in an autocrine manner. The focus of the present review is to summarize the current knowledge of the role of Cx43 and Panx1 in endothelial cells and leukocytes in the vasculature during acute inflammation and to discuss relevant molecular mechanisms regulating Cx43 and Panx1 function.

## Introduction

During acute inflammation the recruitment of leukocytes from the bloodstream to the site of injury or infection is a highly regulated mechanism to ensure that leukocytes leave the bloodstream specifically at the inflammatory site to eliminate the inflammatory trigger (Nourshargh and Alon, 2014). In a series of different consecutive steps including rolling, adhesion processes, crawling, and transmigration through the microvascular endothelial layer leukocytes are in close contact to the endothelium (Nourshargh and Alon, 2014). For a correct regulation of these events during the recruitment cascade, the interaction and communication between leukocytes and endothelial cells (EC) but also between leukocytes or between EC are of particular importance.

Gap junction channels (GJ) are formed by two hemichannels, composed of six connexin (Cx) proteins, and allow the direct transfer of molecules between adjacent cells in a tissue (Kumar and Gilula, 1996). Cx-hemichannels can act as a transmembrane conduit between the cytoplasm and the extracellular space to release paracrine or autocrine signaling molecules (Bruzzone et al., 2001; De Vuyst et al., 2007; Willebrords et al., 2016). Under physiological conditions they are normally closed but open with injury and inflammation and can induce cytokine release (Cheng et al., 2014). Pannexins (Panx) form functional membrane channels and are like Cx-hemichannels and GJ permeable to small molecules including ATP, ions or metabolites (Bruzzone et al., 2003; Zhou et al., 2019). Panx mediated ATP-release has been implicated in inflammatory responses of several immune cells (Lohman et al., 2015; Zhou et al., 2019). Cx and Panx are widely expressed in immune cells and are critically involved in immune cell functions and the regulation of inflammatory responses (Oviedo-Orta and Howard Evans, 2004; Glass et al., 2015; Esseltine and Laird, 2016; Aasen et al., 2019). Among them connexin 43 (Cx43) and pannexin 1 (Panx1) are present in all immune cells and the most studied isoforms (Esseltine and Laird, 2016; Valdebenito et al., 2018; Wang and Chen, 2018). In this mini review we will address the role of Cx43-hemichannels, Panx1-channels as well the intercellular communication via GJ in EC and leukocytes during acute inflammation.

## Impact of Panx1 and Cx43 for Communication of EC and Different Types of Leukocytes (See Figure 1)

Intercellular communication between various cells of the immune system has been described for many years (Hulser and Peters, 1972) and in the last decades the expression pattern of Cx as well as the coupling between immune cells or with EC was analyzed. Also the role of endothelial Cx/GJ for the behavior of leukocytes was focus of many studies (Oviedo-Orta and Howard Evans, 2004; Okamoto and Suzuki, 2017). Intercellular coupling between lymphocytes expressing Cx40 and Cx43 (Oviedo-Orta et al., 2000) was involved in the proliferation of T-cell subsets (Zhang et al., 2018) and lymphocyte-macrophage-coupling (Bermudez-Fajardo et al., 2007). Other leukocytes like neutrophils express various Cx, among them Cx43 (Jara et al., 1995) and are able to form functional GJ among themselves (Branes et al., 2002).

**FIGURE 1:**
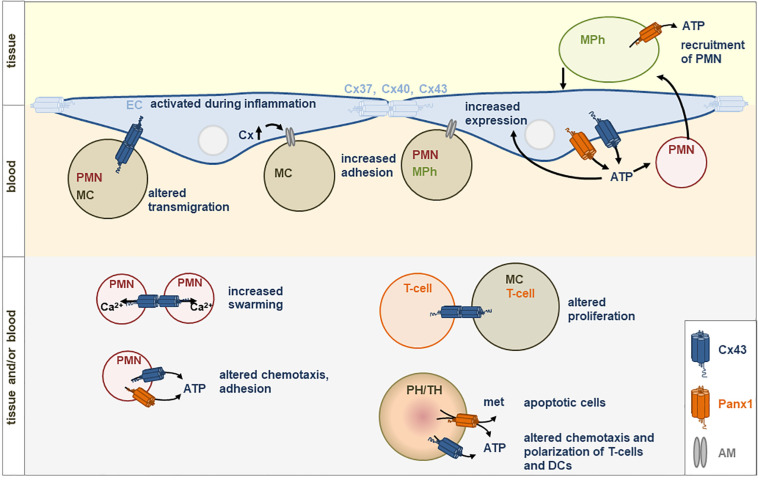
Direct (gap junctions) or indirect (via hemichannels) communication between immune cells (PH, phagocytes; TH, thymocytes; PMN, neutrophils; MC, monocytes; MPh, macrophages) or with endothelial cells (EC) is involved in immune responses. Gap junctions are built by connexins (Cx). Hemichannels can be formed by Cx43 or Panx1. met, metabolites; PIH, pregnancy induced hypertension; AM, adhesion molecules; ATP, Adenosine triphosphate.

Also direct or indirect communication of leukocytes with other cell types like endothelial (Zahler et al., 2003), epithelial (Pepin et al., 2020), or cancer cells (Hofmann et al., 2019) has been detected. Zahler et al. (2003) analyzed neutrophils that were able to exchange a GJ permeable dye with EC and Sarieddine et al. (2009) could demonstrate that adhesion of neutrophils was regulated by gap junctional coupling via Cx43 with endothelial (and epithelial) cells in the lung. Direct communication between lymphocytes is suggested to play a role in T-lymphocyte proliferation contributing to hypertensive inflammatory response (Ni et al., 2017) and GJ between cytotoxic T-lymphocytes and cancer cells allowed the immune system to control cancer cells (Hofmann et al., 2019). Furthermore, Cx43 expression and GJ communication were shown to be transiently induced between monocytes/macrophages at inflammatory sites (Eugenin et al., 2003) and macrophages were shown to communicate via GJ with epithelial cells thereby amplifying the antiviral response (Pepin et al., 2020).

Li et al. (2019) confirmed the role of Cx43 in promoting the adhesion of monocytes to EC. In contrast to others, the authors did not find any adhesive effects of the GJ but could show that enhanced expression of Cx43 in EC led to activation of the PI3K/AKT/NF-κB signaling pathway finally enhancing VCAM-1 and ICAM-1 expression and thereby increasing monocyte–endothelial adhesion. This effect seems to be prominent in EC exposed to high blood pressure leading to an enhanced expression of Cx43 and therefore to an enhanced monocyte-endothelial adhesion. Furthermore, Cx43 expression in monocytes also affects monocyte-endothelial adhesion (Ji et al., 2019) as inhibition of Cx43 expression in monocytes led to a decreased activity of the PI3K/AKT/NF-κB signaling pathway resulting in a decreased monocyte-endothelial adhesion, finally offering new thoughts for understanding atherosclerosis. The release of IL-17 from lymphocytes led to a decrease of Cx43 expression in limbal vascular EC (Qin et al., 2019) indicating an indirect communication of lymphocytes with EC.

Panx can similar to Cx act as hemichannels and were described to be able to form intercellular channels (Bruzzone et al., 2003; Sohl et al., 2005). However, most publications describe their function as hemichannels (Lohman et al., 2015) suggesting an indirect coupling of cells via the release of signaling molecules affecting regulatory processes of effector cells. Panx expression in EC and during inflammation has been reviewed recently (Begandt et al., 2017) and although Panx are known to be important in this context, their regulation in inflammation is unclear (Begandt et al., 2017). However, post-translational modifications of Panx1 (phosphorylation, S-nitrosylation, or caspase cleavage) can affect channel properties (Boyce et al., 2018). As demonstrated in smooth muscle cells a direct Src-dependent phosphorylation at Tyr198 within a Src homology 2 (SH2) domain in the intracellular loop seems to be involved in Panx1-dependent regulation of adrenergic mediated vasoconstriction (Delalio et al., 2019). Moreover, it was shown that spironolactone, an anti-hypertensive drug, inhibits the permeability of Panx1-channels thereby regulating α-adrenergic vasoconstriction (Good et al., 2018).

Under ischemic (Kaneko et al., 2015) or pro-inflammatory (Lohman et al., 2015) conditions, the latter only in venous but not arterial EC, the permeability of endothelial Panx1-channels was increased leading to decreased leukocyte adhesion (Lohman et al., 2015). Furthermore, the opening of Panx1 seems to be involved in ischemia/reperfusion injury-induced inflammation in the brain (Thuringer et al., 2015; Wei et al., 2015) and the lung increasing next to others vascular permeability and neutrophil infiltration (Sharma et al., 2018). Other inflammatory mediators like thrombin or histamine also led to an increased permeability of Panx1 whereas Cx43-hemichannel activity was reduced in this context (Godecke et al., 2012). Next to EC, Panx have also been shown to be expressed in leukocytes (Schenk et al., 2008; Woehrle et al., 2010; Li et al., 2018) and their expression was upregulated due to infection (Li et al., 2018). However, not only the expression was upregulated but also its distribution pattern within the cell changed due to activation of the immune system. In primary T-cells Panx1 was distributed homogenously via the cell surface and translocated rapidly to the immune synapse after stimulation of the cells (Woehrle et al., 2010).

## Cx43 in Leukocyte Recruitment During Acute Inflammation

Leukocyte recruitment during acute inflammation seems to be highly reliant on endothelial Cx43. TNFα-induced leukocyte adhesion and transmigration *in vivo* were reduced in endothelial Cx43-deficient mice as well as in the presence of GJ blockers in wild-type animals (Veliz et al., 2008). In a model of acute lung injury Cx43 was markedly enhanced in the lung of mice subjected to instillation with LPS and neutrophil recruitment was up-regulated in homozygous Cx43^+/+^ compared to heterozygous Cx43^±^ mice (Sarieddine et al., 2009). *In vitro* studies confirmed a reduced adhesion of activated neutrophils to EC or alveolar epithelial cells with blocked Cx43-containing GJ, which are both involved in LPS-induced lung inflammation. In contrast, incubation of GJ-blocked neutrophils with untreated EC did not reduce the number of adherent neutrophils (Sarieddine et al., 2009) indicating that a signal transfer between endothelial or epithelial cells via Cx43-containing GJ significantly contributes to neutrophil recruitment during acute inflammation. In contrast, Cx40 expression is downregulated during acute lung injury and endothelial-specific deletion of Cx40 resulted in increased transmigration of neutrophils after intratracheal instillation of LPS. Downregulation of Cx40 in EC *in vitro*, which was accompanied by a decreased expression and activity of CD73, increased adhesion of neutrophils to the endothelial monolayer suggesting an anti-inflammatory protective role in contrast to Cx43 (Chadjichristos et al., 2010; Scheckenbach et al., 2011). In another study it was shown that treatment of EC with peptidoglycan, a gram-positive bacterial cell wall component, induced ATP-release via Cx43-hemichannel opening which elicited a pro-inflammatory response by induction of interleukin 6 and Toll-like receptor 2 expression (Robertson et al., 2010), both known to be involved in leukocyte recruitment (Jones, 2005; Sabroe et al., 2005).

In neutrophils, Cx43 is primarily involved in ATP-release via hemichannels (Eltzschig et al., 2006). Dependent on the extracellular ATP concentration different functions like chemotaxis or adhesion are affected by different purinergic receptors (Trautmann, 2009; Wang and Chen, 2018). The ATP-release via Cx43-hemichannels from activated neutrophils during acute inflammation may have endothelial barrier protective and anti-inflammatory effects (Eltzschig et al., 2006). Phosphorylation of Cx43 at Ser368 is associated with reduced opening (Bao et al., 2004) and ATP-release of activated neutrophils is regulated by Ser368 de-phosphorylation via phosphatase A. This phosphorylation-dependent ATP-release decreased endothelial paracellular permeability by modulating endothelial adenosine responses and might serve as a stopping signal for neutrophil chemotaxis (Eltzschig et al., 2006) to halt their migration at the infectious site. Moreover, it has been shown that LPS-induced autocrine ATP signaling inhibited neutrophil chemotaxis and knockout of the ATP receptor (P2X1) in neutrophil-like differentiated HL-60 cells recovered chemotaxis (Wang et al., 2017). LPS-induced ATP-release through Cx43-hemichannels of activated neutrophils induced a calcium ion (Ca^2+^) influx via P2X1 receptors, a ligand-gated cation channel with high Ca^2+^ permeability, which reduced neutrophil chemotaxis by activating myosin light chain phosphorylation (Wang et al., 2017). Of note, degranulation and phagocytosis were enhanced by autocrine ATP-release. Hence, activated neutrophils release ATP to stop chemotaxis which might therefore facilitate the bactericidal functions of neutrophils (Wang et al., 2017). Moreover, neutrophil Cx43 has been shown to be crucial for coordinated Ca^2+^ fluxes and initiation of the neutrophil swarming (Poplimont et al., 2020). The first step, the initial recruitment of neutrophils to the site of inflammation is induced by damage-associated molecular patterns (DAMPs) from damaged or dead cells and pathogen-associated molecular patterns (PAMPs) released by invading microorganisms, causing the release of chemo-attractants by local tissue cells. In contrast, the second step, the highly coordinated neutrophil movement with dense clusters and swarm aggregation at the wound core is mediated by paracrine release of the chemoattractant leukotriene B4 (LTB4), whose synthesis requires a sustained Ca^2+^ flux (Kienle and Lammermann, 2016; Poplimont et al., 2020). Cx43-dependent ATP-release via hemichannels induced calcium signal propagation in clustering neutrophils leading to LTB4 biosynthesis in neutrophils in a zebrafish model (Poplimont et al., 2020). The authors conclude that hemichannel opening of pioneer neutrophils upon contact with death-specific signals such as fMLP mediate Ca^2+^ flux-dependent LTB4-release, activating a secondary Cx43-channel opening leading to sustained intracluster Ca^2+^ fluxes (Poplimont et al., 2020). This study indicates that Cx43-channels play a crucial role for neutrophil swarm generation and chemoattractant production during acute inflammation.

The motility and transendothelial migration of B-cells also depend on Cx43 and are decreased after downregulation, likely regulated channel-independently by Cx43 (Machtaler et al., 2014), indicating that Cx43 can affect the individual steps of leukocyte recruitment via different mechanisms.

## Panx1 in Leukocyte Recruitment During Acute Inflammation

Panx can be considered as key upstream molecules in inflammasome activation (Pelegrin and Surprenant, 2006) and several studies have shown that ATP-release through Panx1-channels enhances inflammatory responses (Adamson and Leitinger, 2014). The progressive opening of Panx1-channels in response to inflammatory stimuli can be activated by a caspase-dependent cleavage of the C-terminal tail controlling channel permeability (Makarenkova et al., 2018; Chen et al., 2019) associated with NLRP3 inflammasome activation in apoptotic macrophages (Chen et al., 2020). Moreover, Panx1 promotes recognition of bacterial molecules to activate the cryopyrin inflammasome (Kanneganti et al., 2007). The interaction of Panx with P2X_7_ receptors has been reported to create a large permeable pore at the cell membrane and ATP-mediated activation of the inflammasome promotes the secretion of cytokines (Adamson and Leitinger, 2014). The release of ATP via Panx1-channels is involved in leukocytes recruitment by guiding chemotaxis of neutrophils and macrophages via purinergic receptor signaling activated by extracellular ATP (Adamson and Leitinger, 2014). Studies on neutrophil migration showed that ATP-release via Panx1-channels plays a dual role to trigger signals: excitatory signals by amplifying chemotactic signals and facilitating gradient sensing at the front of polarized neutrophils and inhibitory signals via A_2A_ adenosine receptors involved in blocking chemoattractant receptor signaling at the back of migrating neutrophils, demonstrating the role of Panx1 as fine-tune regulator of chemotactic responses of neutrophils (Chen et al., 2006, 2010; Bao et al., 2013). The ATP-release via Panx1-channels and autocrine stimulation of P2X_4_ receptors also contributes to T-cell polarization and migration, whereas blocking of P2X_4_ receptors inhibited T-cell activation and migration *in vitro* (Ledderose et al., 2018). ATP-release via Panx1 is also required for secretion of IL-1β from macrophages induced by P2X_7_ receptor activation (Pelegrin and Surprenant, 2006). In dendritic cells extracellular ATP stimulated fast cell migration through an autocrine signaling loop initiated by P2X_7_ receptor activation and amplified by opening of Panx1-channels and supplementary release of ATP (Saez et al., 2017).

The extravasation of leukocytes across the vascular wall to the site of injury is dependent on Panx1-mediated ATP-release from venous endothelium (Lohman et al., 2015). Stimulation of venous EC with TNFα induced expression and activation of Panx1-channels stimulating Ca^2+^-dependent synthesis of IL-1β (Yang et al., 2020) and ATP-release inducing leukocyte adhesion and emigration *in vitro* and *in vivo* (Lohman et al., 2015). The latter might be partially mediated by increased endothelial expression of the adhesion molecule VCAM-1 (Dosch et al., 2018). Notably, EC derived nitric oxide can inhibit Panx1 activity through S-nitrosylation which reduces ATP-release and Panx1-channel currents in murine EC (Lohman et al., 2012).

Recently, Tam et al. (2020) demonstrated that during metabolic inflammation pro-inflammatory macrophages release chemotactic factors upon exposure to saturated fatty acids, such as palmitate, which was associated with recruitment and infiltration of neutrophils into adipose tissues. Heat inactivation or proteinase K digestion of media released by fatty acid treated macrophages did not alter neutrophil recruitment but was effective to inhibit CXCL-1 induced chemotaxis suggesting that these macrophage derived chemotactic factors are not proteins (Tam et al., 2020). Moreover, the authors found that palmitate upregulated Panx1-channel expression in macrophages mediating neutrophil attraction by release of nucleotides such as ATP. The thereby following purinergic receptor stimulation and signaling induces neutrophil migration (Tam et al., 2020). These results emphasize the role of Panx1 in neutrophil recruitment and its contribution to inflammatory response during obesity. The impact of Panx1 for leukocyte recruitment during inflammation has also been demonstrated using a pharmacologic blocking of Panx1-channels by trovafloxacin, which decreased ICAM-1 expression and delayed recruitment of monocytes and neutrophils (Giustarini et al., 2019).

Due to the fact that Panx1-channels also regulate host responses to viruses (Orellana et al., 2013; Malik and Eugenin, 2019) the impact of Panx1 in COVID19 patients displaying hyper-inflammation has recently been discussed (Swayne et al., 2020). For the patient recovery especially the early phase of innate immunity seems to be critical and immunosuppression could limit viral entry and disease progression. Therefore, targeting Panx1-channels could improve therapeutic approaches and might reduce SARS-CoV-2 infectivity and vascular inflammatory responses in COVID19 patients (Swayne et al., 2020).

## Molecules Transmitted via Panx1-Channels and Cx43-Channels

There are many data about the release of ATP via Cx43- or Panx1-channels: neutrophils for example are known to release ATP via Cx43-hemichannels during hypoxia or inflammation (Eltzschig et al., 2008). In inflamed lung EC, the release of ATP was shown to be mediated via Panx1 (Sharma et al., 2018) and in a fish model tPanx1-channels in leukocytes and other cells were responsible for the release of ATP (Li et al., 2018). In smooth muscle cells Panx1 mediated ATP-release is regulated by a direct Src-dependent phosphorylation at Tyr198 (Delalio et al., 2019) and purinergic regulation of migrating T-cells was shown to be stimulated by an ATP-release via Panx1 (Ledderose et al., 2018). For intercellular communication via GJ, it is common knowledge that molecules up to a size of 1.8 kD can be transferred between cells (Neijssen et al., 2005). Typically, ions like K^+^ or Ca^2+^ and second messengers like cAMP were shown to diffuse from one cell to the other (Valiunas et al., 2019) but for the communication between either immune cells or between immune and endothelial/epithelial cells less data are available. Zhang et al. speculated that ATP diffuses via GJ between lymphocytes (Zhang et al., 2018). For cytotoxic T-cells that form GJ with cancer cells leading to an enhanced granzyme B activity in cancer cells it was thought that granzyme B or Ca^2+^ may be transferred (Hofmann et al., 2019) and macrophages that communicate via GJ with epithelial cells (Pepin et al., 2020) seem to mediate cGAMP via intercellular channels. Also peptides have been shown to be transferred via GJ between cells causing recognition of nearby bystander cells and activated monocytes by cytotoxic T-cells (Neijssen et al., 2005).

## Discussion

The activity of both Cx43-hemichannels and Panx1-channels depends on diverse environmental stimuli and increases under pathophysiological conditions including acute inflammation. Their opening allows the release of signaling molecules from the cytoplasm acting as autocrine or paracrine messengers (Evans et al., 2006) and enables activated EC and leukocytes to fine-tune responses during acute inflammation. Most of the described effects of inflammatory cells are mediated via ATP-release through Cx43-hemichannels or Panx1-channels and purinergic receptor signaling but also the gap junctional transfer of signaling molecules affects the recruitment of leukocytes. Despite the increasing amount of studies on Cx- and Panx-functions in EC and leukocytes still little is known about their interplay and regulation during acute and chronic inflammation and requires further investigations. Moreover, it should be mentioned that pharmacologic inhibition of Cx43-hemichannels at least partly affects Panx1 and GJ and making the discrimination between hemichannels and GJ difficult (Willebrords et al., 2017). However, targeting Panx1- or Cx43-dependent ATP-release and purinergic signaling could represent a promising approach for the therapeutical treatment of acute inflammation.

## Author Contributions

PK and KP wrote the manuscript. Both authors contributed to the article and approved the submitted version.

## Conflict of Interest

The authors declare that the research was conducted in the absence of any commercial or financial relationships that could be construed as a potential conflict of interest.
